# Post-Anæsthetic Broncho-Pneumonia
*The preceding article appeared last week.


**Published:** 1912-02-24

**Authors:** 


### POST-ANÆSTHETIC BRONCHO-PNEUMONIA.*

### II.—Some Points Regarding its Treatment.

The treatment of post-anæsthetic pneumonia may fitly be considered under two headings—namely, *prophylactic treatment* and *treatment during the attack*. The former is by far the better line of treatment, and resolves itself, when all is said and done, into the precautionary measures which should be taken in every case in which an anæsthetic, no matter of what kind, is rendered necessary in view of operative or other interference.

### Prophylactic Treatment.

*Before the Administration of the Anæsthetic.*—Care should be taken to examine the patient thoroughly. This is generally done by the house surgeon or physician in a hospital case, and by the private medical attendant in a private case. Nevertheless, the anæsthetist should not be content merely to accept their findings, but should examine the patient for himself. Percussion and auscultation should be employed in examining the chest; the nose and mouth should be inspected, and especial care should be taken in the case of subjects who are emphysematous or bronchitic, in listening to the sounds at the lung bases. Very often slight crepitations and a few isolated rhonchi are heard over the bases in patients who reveal no abnormal sounds over the upper lobes. Definite bronchitis is, of course, no contra-indication to the administration of a general anæsthetic any more than a definite heart murmur is a contra-indication, but both call for close attention on the part of the anæsthetist in selecting and in administering the anæsthetic. Where a marked capillary bronchitis exists in a patient who is old or enfeebled, it is generally better to give a spinal injection or to defer the operation, if possible, until the bronchial condition is bettered. Where lumbar anæsthesia is out of the question—as in a case of marked sepsis—and where the operation cannot be put off, extra precautions must be used. The patient's mouth, also, needs attention. Septic and decayed teeth should be removed or stopped; if that is impossible, a good alkaline mouth-wash must be ordered to be used some days before the operation; in an emergency, the patient must be made to rinse his mouth thoroughly with an antiseptic mouth-wash, brush his teeth, and generally disinfect his mouth and nose. Of not less importance is it to ensure that the patient is warm and comfortable during the night, or at least during the period immediately preceding the operation. If a purgative or enema is given the bed-pan must be used, and the patient must not be allowed to visit the closet or use a night-commode, as this invariably subjects him to exposure. His underclothing and night-shirt should be warm; bed hose should be worn, and a hot-water bottle placed in his bed. Starvation before an anæsthetic is a barbarous and unnecessary safeguard, which is now generally condemned. All that is necessary is to see that the patient's bowels are clear and that his stomach is not overloaded. A light and nourishing meal given a few hours before the operation is, therefore, advisable. Even where the stomach has to be washed out immediately before the operation, such a meal is useful and advisable. Nutrient enemata are probably of very little use: a glucose solution injected into the lower bowel is perhaps the best form of this kind of enema that can be given. Whether a preliminary hypodermic injection of scopolamine, omnopon, salipin, or some other drug is necessary will depend largely on the faith which the surgeon or anæsthetist has in these adjuvants, the use of which we have already discussed. Similar precautions must be taken to guard against chill when moving the patient to and from the operating-room. Especial care is necessary during the removal from the theatre to the bed. The vitality of the patient is then low, and the change from the often overheated atmosphere of the theatre to the generally colder air in the corridors and bedroom is necessarily predisposing to a chill. Warm blankets, hot-water bottles, and good covering of the extremities will obviate such a contingency.

### Some Needful Precautions.

*During the Administration of the Anæsthetic.*—The choice of the anæsthetic will depend on the condition of the patient; that is the only consideration that matters. The wish of the surgeon, the predilection of the anæsthetist, and the prejudice of the patient himself (who often displays a marked objection to one or other anæsthetic) must be subservient to it. We need not here discuss the question of choice, as it has already been sufficiently elaborated in the first article. Whatever anæsthetic is chosen care must be taken that the patient is never exposed unduly, that a sufficient airway is left, that the vapour is not below body temperature—a point which is of special importance when compressed oxygen or gas is administered—that the mouth is kept free of saliva, and that the head be kept lowered. Where the Trendelenburg or the lithotomy position is used, caution should be exercised when the alterations in position are made; all sudden lowering or elevating must be avoided and the position of the patient changed gradually. An even degree of anæsthesia should be maintained, and the minimum amount of anæsthetic necessary to keep up this degree should be used. The induction must be gradual, and coughing and swallowing must be avoided. In short, the ordinary precautions which every competent anæsthetist will be acquainted with must be taken.

When the operation is over and the patient is back in bed, he must be watched to see that he does not throw the bedclothes from him. Early deep inspiration should be encouraged, especially in an abdominal case; post-anæsthetic vomiting and nausea must be properly dealt with. A more or less elevated position of the upper part of the patient's body, in every case where pulmonary complications exist, is desirable; where bronchitis exists it must be actively treated.

* The preceding article appeared last week.

The onset of post-anæsthetic broncho-pneumonia proclaims itself by the rise in the temperature, and in the pulse rate, and in the onset of distressing cough and the loss of spirits and animation on the part of the patient. Such onset is not usually delayed much beyond 24 hours after the administration of the attack; cases that develop later are generally more severe than those which show themselves within 24 hours.

PREMONITORY SYMPTOMS.

One of the earliest signs is the depression of spirits and feeling of malaise which the patient complains of. He may have awakened from the operation feeling merely a little weak, with no pain or inconvenience, and may have spent the first ten hours cheerfully, only, later on, to become restless and to complain of cough and slight thoracic pain. On examination the usual signs are discovered. The sputum is frothy, at first whitish grey, later on purulent and blood-stained or rusty; there is impairment of the percussion node over the area of lung affected, and over the same area signs of congestion and consolidation are apparent on listening. The examination of the chest in patients who show any rise of temperature or quickening of the pulse rate, even when they do not complain of cough or thoracic pain, should be systematic and thorough; only by this means is incipient broncho-pneumonia discovered. And the earlier its presence is definitely diagnosed the better, owing to the fact that the disease, in the milder cases at least, yields very readily to treatment.

It must be remembered that we are now dealing with post-anæsthetic pneumonia, not with the more severe form of the same disease known as aspiration, surgical, septic, or post-operative pneumonia, which is much more fatal, and which usually develops two or three days or even later after the operation. The active treatment of post-anæsthetic pneumonia consists mainly in allaying the distressing cough, which generally strains the wound and renders the patient extremely uncomfortable, and in treating the symptoms as they arise. The disease itself must be allowed to run its course; no remedy has any direct effect upon it, but if the patient's strength is kept up, the course of the pneumonia is generally soon checked. Perhaps the most comforting line of treatment to the patient is to apply moist heat to the thorax. A flannel compress, wrung out in hot water and arranged with a covering of gutta-percha or christia tissue, serves excellently, but must be frequently renewed. An electric compress—a flannel or felt bag warmed by electric wires—is more convenient; care must be taken that the flannel does not get wet by perspiration, else short-circuiting may result and the patient may get badly burned. Antiphlogistine, applied in a fairly thick layer, has lately been highly recommended; it is certainly a very soothing and convenient application and does not need renewal for several days.

Against the cough opium in some form or other is the best remedy. A linctus of syrup of codeine with syrup of Virginia prune a.a. in drachm doses is one of the best remedies to prescribe, but the choice of prescriptions is so large that it is somewhat difficult to make a selection. Sedatives must be chosen instead of stimulating expectorants. When the disease progresses in spite of treatment it may be necessary to administer cardiac stimulants; of these caffeine, camphor, musk, strychnine, and, best of all, alcohol may be tried. The patient's strength must be maintained by good food of an easily digestible nature. He must be allowed the maximum amount of fresh air, care being taken to avoid draughts.

When the attack subsides breathing exercises are desirable, but the best way to hasten convalescence is to remove the patient to the seaside or to a place where he can have the benefit of pure upland country air. After a severe attack patients are, as a rule, much debilitated and greatly in need of continuous tonic treatment for some weeks. A mild attack which spends itself in a couple of days may, on the other hand, not greatly interfere with convalescence.

